# Reserve lipids and plant autophagy

**DOI:** 10.1093/jxb/eraa082

**Published:** 2020-02-21

**Authors:** Céline Masclaux-Daubresse, Sabine d’Andrea, Isabelle Bouchez, Jean-Luc Cacas

**Affiliations:** 1 Institut Jean-Pierre Bourgin, INRAE, AgroParisTech, Université Paris-Saclay, Versailles, France; 2 University of Essex, UK

**Keywords:** Autophagy, beta-oxidation, endoplasmic reticulum, leaf senescence, lipidomic, lipid droplet, lipids, oil body, oleosome, triacylglycerol

## Abstract

Autophagy is a universal mechanism that facilitates the degradation of unwanted cytoplasmic components in eukaryotic cells. In this review, we highlight recent developments in the investigation of the role of autophagy in lipid homeostasis in plants by comparison with algae, yeast, and animals. We consider the storage compartments that form the sources of lipids in plants, and the roles that autophagy plays in the synthesis of triacylglycerols and in the formation and maintenance of lipid droplets. We also consider the relationship between lipids and the biogenesis of autophagosomes, and the role of autophagy in the degradation of lipids in plants.

## Teaser

We review recent developments that suggest that autophagy plays a role in lipid homeostasis in plants in a manner that is comparable to algae, yeast, and animals.

## Introduction

Autophagy (‘self-eating’) is a universal mechanism that facilitates the degradation in eukaryotic cells of unwanted cytoplasmic components. Two distinct types, micro- and macro-autophagy, have been reported to play roles in metabolism and nutrient recycling in plants. Micro-autophagy is characterized by the invagination of the tonoplast to trap cytoplasmic material and create autophagic bodies within the vacuole. Macro-autophagy involves the formation in the cytosol of double-membrane vesicles, called autophagosomes, that sequester cytoplasmic components and carry them to the lytic vacuoles where they are released. Up to 40 autophagy genes (ATGs) are involved in the macro-autophagy machinery; 18 of them constitute the core machinery that participates in the formation of autophagosomes. By contrast with macro-autophagy, the molecular and genetic bases of micro-autophagy remain unclear. Links between macro- and micro-autophagy and plant primary and secondary metabolism have been documented in several reports. In this review, we highlight recent developments in the investigation of the role of autophagy in lipid homeostasis in plants by comparison with algae, yeast, and animals.

## Lipid sources in plants

As in other eukaryotic cells, lipids are found in membranes but are also stored as droplets in the plant cell. Triacylglycerols (TAGs) packed in lipid droplets (LDs) represent a major form of carbon storage. Oleaginous plants such as rapeseed, soybean, sunflower, and peanut accumulate numerous LDs in their seeds. Other plants such as avocado and olive store lipids in the pericarp of their fruits. Bio-membranes consist of double layers of polar lipids that are mainly composed of glycerolipids (phospholipids and galactolipids). Membranes also contain sterols and long-chain sphingolipids (Gomez *et al.*, 2018). While galactolipids in plants are located in the membranes of chloroplasts, sphingolipids such as glycosyl inositol phosphorylceramide (GIPC) are preferentially localized to the plasma membrane where they are highly enriched in microdomains, together with sterols (Cacas *et al.*, 2016).

LDs are formed of apolar lipids (i.e. TAGs and sterol esters) surrounded by a monolayer of phospholipids containing specific proteins. The LDs of the seeds are also referred to as oleosomes, as they are coated by a specific family of proteins called oleosins. The presence of oleosins at the surface of the seed LDs prevents the phospholipids from adjacent LDs coming into contact and fusing with them (Schmidt and Herman, 2008; Miquel *et al.*, 2014). Although LDs mainly accumulate in the embryo or endosperm of oleaginous seeds, they can also be found in the leaves, especially during senescence and under stressful conditions (Slocombe *et al.*, 2009; Pyc *et al.*, 2017; Coulon *et al.*, 2020). LDs isolated from leaves and oleaginous fruit tissues of Arabidopsis differ from those isolated from the seeds: instead of oleosins they are coated with a family of proteins called LD-associated proteins (LDAPs; also termed small rubber-particle proteins, SRPs) (Horn *et al.*, 2013; Gidda *et al.*, 2016; Brocard *et al.*, 2017; Coulon *et al.*, 2020). Interestingly, whereas the expression of oleosins is developmentally controlled and restricted to seed and pollen, the expression of LDAPs is induced in response to stress (Miquel *et al*., 2014; Gidda *et al.*, 2016). The formation of LDs occurs at the membrane of the endoplasmic reticulum (ER) and is facilitated in leaves by the interaction between LDAP and LDAP-interacting protein (LDIP) (Pyc *et al.*, 2017; Coulon, *et al.*, 2020).

In plants, *de novo* biosynthesis of fatty acids (FAs) takes place in the chloroplast, while TAG assembly occurs at the ER. Newly synthesized FAs are exported from the plastids and eventually modified by desaturation after incorporation into phosphatidylcholine (PC) in the ER. The assembly of FAs into TAGs occurs through different pathways that take place in the ER (reviewed by Li-Beisson *et al.*, 2013). Indeed, both the diacylglycerol acyl transferase DGAT1 that is involved in the last step of the Kennedy pathway and the phospholipid:diacylglycerol transferase PDAT1 that acylates the diacylglycerol (DAG) using PC as the acyl donor reside in the ER (Kaup *et al.*, 2002; Chapman *et al.*, 2013; Brocard *et al.*, 2017). The TAGs accumulate between the two leaflets of the ER membranes to form LDs that bud from the ER and are eventually released into the cytosol.

Both membrane and storage lipids are used to fulfil the β-oxidation pathway and produce energy in the mitochondria of mammals, and in the peroxisomes of yeast and plants. During leaf senescence, peroxisome β-oxidation is enhanced (Charlton *et al.*, 2005; Yang and Ohlrogge, 2009; Watanabe *et al.*, 2013; Zhang *et al*., 2020) and the accumulation of LDs suggests that the FAs released from the membranes are first interconverted as TAGs in droplets to prevent FA-induced toxicity before being used for β -oxidation (Kaup *et al.*, 2002; Lin and Oliver, 2008; Yang and Ohlrogge, 2009). The TAGs and lipid-esters can also accumulate in the chloroplasts during senescence, forming plastoglobules. These are formed from the FAs released from the degradation of the galactolipids and phytols (Lippold *et al.*, 2012). The dynamics of the formation and degradation of LDs in vegetative plant tissues are yet to be determined.

## Does autophagy play a role in TAG synthesis and in the formation and maintenance of LDs in cells?

Whether or not autophagy is involved in the formation and/or maintenance of LDs has been questioned (see Elander *et al.*, 2018, for a review). TAGs are less abundant and LDs are smaller in the cells of the *atg8* yeast mutant compared to the wild-type at the stationary phase (Maeda *et al.*, 2017); conversely, higher levels of TAGs and larger LDs are found in *Atg8* overexpressors. This led the authors to propose that a specific function of Atg8 in yeast may be to maintain the quantity of LDs; however, this was probably independent of the function of Atg8 in autophagy.

Nitrogen and phosphate starvation are known to enhance the formation of LDs in *Chlamydomonas reinhardtii.*  Treatment with concanamycin A, which inhibits the degradation of cargoes in the vacuole, prevents the formation of LDs and the synthesis of TAGs in nitrogen- and phosphate-limited cells (Couso *et al.*, 2018). This suggests that autophagy plays a role in the biogenesis of TAGs and LDs in *C. reinhardtii*.

In mammals, the differentiation of adipocytes correlates with the enhancement of autophagic flux. Conversely, mutations in the autophagy pathway abolish the accumulation of TAGs and impair adipocyte formation (Baerga *et al.*, 2009; Zhang *et al.*, 2009). Providing oleate to mammalian cells induces the formation of LDs, and in turn increases autophagic flux. In the absence of a build-up of LDs, autophagy still occurs in mammalian cells but at a lower rate. Transient interactions between LDs and LC3 (the ATG8 homologue in mammals) have been demonstrated (Singh *et al.*, 2009; Shibata *et al.*, 2009; Dupont *et al*., 2014*a*, 2014*b*). DGAT1 resident in the ER has been shown to selectively channel the FAs liberated by autophagy into new LDs that cluster in close proximity to mitochondria (Nguyen *et al.*, 2017; Nguyen and Olzmann, 2017).

In rice, the *OsATG7*-knockout mutant *osatg7*, which is defective in autophagy, displays male sterility and lower contents of TAGs and LDs in mature anthers compared to the wild-type (Kurusu *et al.*, 2014). Defects in phosphatidylcholine synthesis and lipid desaturation during pollen maturation may explain the male-sterility phenotype of *osatg7*. Fan *et al.* (2019) showed that TAGs levels are lower in the leaves of the Arabidopsis *atg2* and *atg5* mutants compared with the wild-type. Using ^14^C-acetate and ^3^H_2_O short-term labelling, they demonstrated that the decrease of TAG synthesis in the autophagy mutants was not due to a decrease in the rate of neo-synthesis of FAs but was instead due to a decrease in the turnover of membrane lipids. Minina *et al*. (2018) showed that the concentrations of FAs in the seeds of the Arabidopsis *atg7* and *atg5* mutants are not different from those of the wild-type; however, overexpression of *ATG5* or *ATG7* under the control of the 35S promoter increases the concentration of FAs, probably due to better allocation of resources.

Taken together, these studies indicate a link between autophagy and lipid metabolism in yeast, algae, animals, and plants.

## Lipids in the biogenesis of autophagosomes

It has long been known that the formation of autophagosomes requires the recruitment of lipids to the pre-autophagosomal structure and this is mediated by the ATG9 transmembrane protein (Orsi *et al.*, 2012; Yamamoto *et al.*, 2012). Recently, Zhuang *et al.* (2017) have shown that ATG9 deficiency in Arabidopsis leads to a drastic accumulation of autophagosome-related tubular structures in direct membrane continuity with the ER. This demonstrates that ATG9 is essential in regulating autophagosome formation from the ER membrane in Arabidopsis, as is the case in animals and yeast.

Although the ER membrane is assumed to be the origin of lipids for autophagosome biogenesis, several lines of evidence show that LDs may also be a source. Shpilka *et al.* (2015) showed in yeast that LDs can provide lipid precursors and phospholipids to the growing autophagosomes, with lipid exchanges occurring at the LD–ER and ER–autophagosome contact sites and *via* interactions between the LDs and the autophagosomes. In mammals, *TMEM41B*-knockout cells accumulate LDs and are autophagy defective (Moretti *et al.*, 2018). TMEM41B is located at the ER membrane and is probably essential for lipid exchanges between the LDs and the pre-autophagosomal membranes. A recent study has shown that autophagosome membranes do not only derive from preformed membranes of organelles or from LDs. Schütter *et al.*, (2020) found that localized phospholipid synthesis controls the expansion of the phagophore membrane in yeast. The acyl-CoA synthetase Faa1, which accumulates locally on nucleated phagophores, activates FAs into the synthesis of phospholipids and promotes their assembly into autophagic membranes. Studies of mutants and the use of specific inhibitors showed that Faa1 is critical for autophagy in yeast. However, it remains to be determined whether the neo-synthesis of phospholipids that is activated by Faa1 is localized at the ER–phagophore contact sites and whether the ATG2–ATG18 complex is involved in the transfer of these phospholipids to expanding phagophores, as for example in mammals (Maeda *et al.*, 2019).

## Autophagy and lipid degradation in plants

**Fig. 1.**
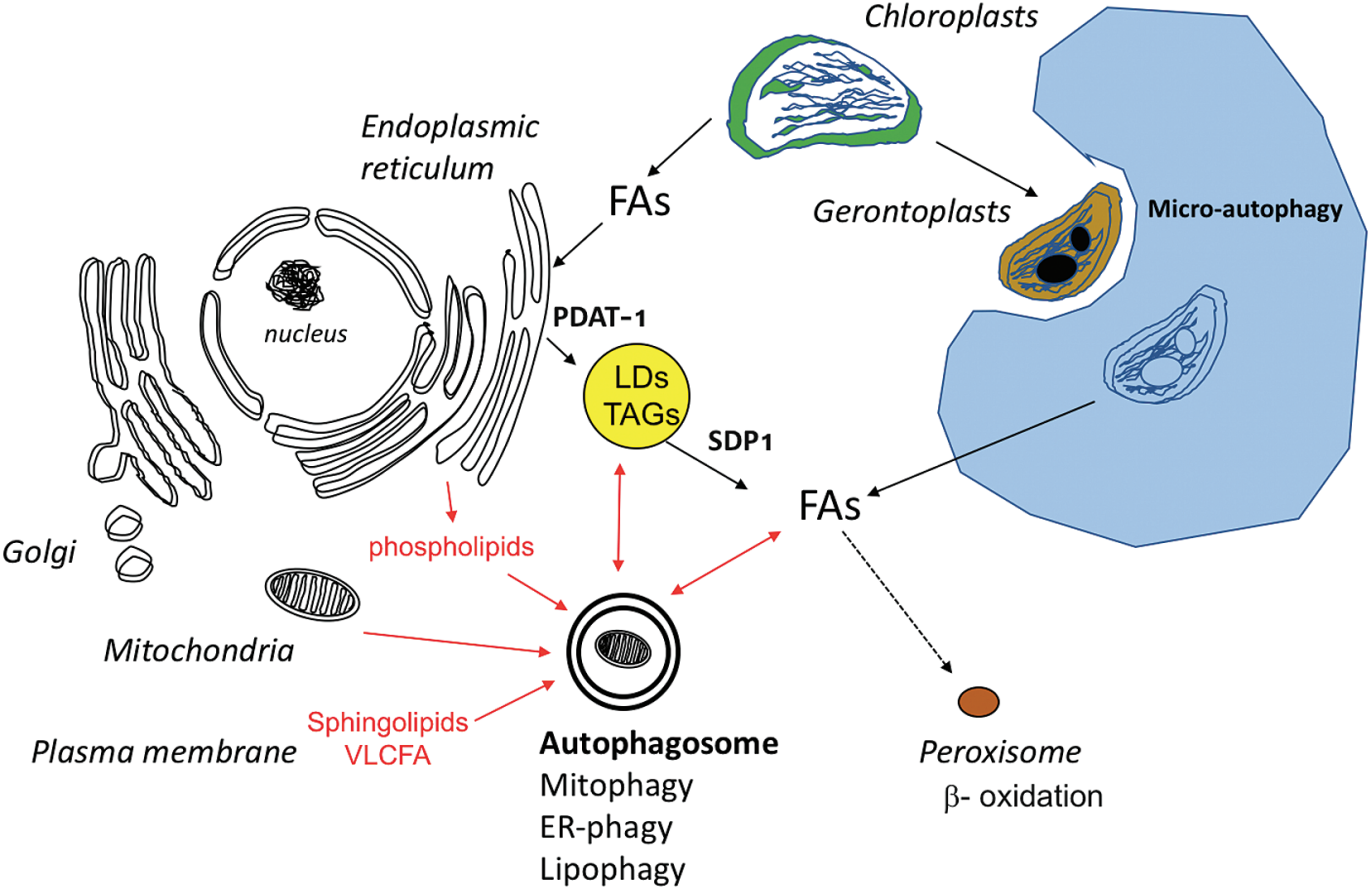
Schematic representation of the potential roles of autophagy in lipid degradation in Arabidopsis leaves. According to Fan *et al.* (2019) and Havé *et al.* (2019), autophagy may be involved in the recycling of lipids that originate from the plasma membrane and the endomembrane of several organelles, except for those of chloroplasts. While the stroma material of the chloroplasts is degraded through the macro-autophagy pathway, chlorophagy occurs through the micro-autophagy pathway (Izumi *et al.*, 2019). Red arrows indicate autophagy-dependent lipid degradation pathways: double-ended arrows indicate that autophagy may be involved in both the degradation and replenishment of lipid droplets and fatty acids. LDs, lipid droplets; TAGs, triacylglycerols; FAs, fatty acids; SDP1, SUGARDEPENDENT1 triacylglycerol lipase; PDAT-1, phospholipid:diacylglycerol transferase, VLCFA, very-long-chain fatty acid; ER, endoplasmic reticulum.

Lipid degradation involving autophagy is referred as lipophagy, and lipid degradation by lipases in the cytosol is referred as lipolysis. Lipophagy can occur by both macro- and micro-autophagy. In algae and yeast both macro- and micro-lipophagy have been observed (Weber and Davoli, 2002; van Zutphen *et al.*, 2014; Wang *et al.*, 2014; Zhao *et al.*, 2014; Schwarz *et al.*, 2017; Kajikawa *et al.*, 2019). In yeast, lipophagy by direct engulfment of LDs into the central vacuole has been reported (Table 1). This micro-autophagy process requires the integrity of most of the proteins of the core macro-autophagy machinery. In animals, the degradation of LDs is mainly achieved by the macro-autophagy machinery. However, there are several lines of evidence that chaperone-mediated autophagy and lipolysis by adipose triglyceride lipase (ATGL) also play a role (Wang *et al.*, 2012; Schulze *et al.*, 2013, 2017; Khaldoun *et al.*, 2014; Kaushik and Cuervo, 2015; Rambold *et al.*, 2015; Schroeder *et al.*, 2015; Martinez-Lopez *et al.*, 2016; Tsai *et al.*, 2017; Demine *et al.*, 2018). In plants, very little is known about the degradation of LDs except that SUGARDEPENDENT1 (SDP1) triacylglycerol lipase is involved in their lipolysis in dark-treated leaves and also in the degradation of seed LDs post-germination (Thazar-Poulot *et al.*, 2015; Fan *et al.*, 2017). Recently, Fan *et al.* (2019) used ^14^C-acetate labelling and performed chase experiments on Arabidopsis mutants deficient in autophagy and on mutants known to enhance TAG synthesis through the ER or chloroplast pathways. The ^14^C labelling in TAGs showed that autophagy participates in the neo-synthesis of FAs in young, growing leaves, and also in the turnover of lipids of the endomembranes in mature and senescing leaves. The authors demonstrated that inactivating autophagy inhibits the mobilization of FAs from the membranes for TAG synthesis. They did this by combining mutations in *ATG5* and *ATG2* first with constructs overexpressing phospholipid:diacylglycerol acyltransferase (PDAT1) and OLEOSIN-1, which enhance formation and accumulation of LDs, second with a mutation in *trigalactosyldiacylglycerol1* (*tgd1*), which impairs the ER lipid pathway and enhances the chloroplast pathway for TAG synthesis, third with a mutation in *SDP1*, and fourth with a mutation in the plastid *trigalactosyldiacylglycerol1* (*act1*), which controls chloroplast FA synthesis. However, using dark treatments, they showed that autophagy has no impact on the synthesis of FAs in the chloroplast and that the recycling of the thylakoid membranes is not modified in the autophagy mutants. Their electron micrographs suggested that the lipophagy of LDs occurs in a resembling micro-lipophagy that requires the macro-autophagy core machinery. Fan *et al.* (2019) concluded that macro-autophagy is involved in the degradation of lipids originating from the endomembranes of various organelles, except for those of the chloroplasts. Interestingly, their results were in good agreement with proteomic and lipidomic studies performed by Havé *et al.* (2019), who used comparisons of the proteomes of Arabidopsis *atg5* and *atg5/sid2* mutants with the Col-0 wild-type and the *sid2* mutant to show that defects in autophagy triggered stress in the ER and increased the abundance of enzymes involved in lipid metabolism in the ER and peroxisomes, irrespective of nitrate or sulphate availability. Enzymes involved in the elongation of very-long-chain FAs together with the PDAT1protein were more abundant in *atg5* and *atg5/sid2* than in the control lines. Enzymes involved in the peroxisome β-oxidation pathway were also more abundant while chloroplast enzymes involved in FA synthesis were less abundant. In parallel with a decrease of chloroplast proteins in the *atg5* mutants, a decrease of galactolipids was also observed. These decreases were more severe under conditions of nitrate and sulphate limitation than under control nutrition, showing that chloroplasts were degraded in the *atg5* lines independently of autophagy when the plants were starved. Many proteases including SAG12 and AED1 are strongly increased in the *atg5* mutant, suggesting that alternative pathways are involved in the degradation of chloroplasts when autophagy is defective (Guiboileau *et al.*, 2013; Havé *et al.*, 2018). By contrast with galactolipids, the *atg5* mutants exhibit accumulation of phospholipids (mainly phosphatidylcholine, phosphatidylinositol, and phophatidic acid) and sphingolipids (long-chain ceramides and hydroxyceramides, and GIPC) compared with control lines. This therefore suggests that the turnover of lipids originating from the ER and plasma membranes is impaired in autophagy mutants. The studies of Fan *et al.* (2019) and Havé *et al.* (2019) consequently both raise the same conclusion that the autophagy pathway is involved in the turnover of the lipids originating from the plasma membrane and organelles, but not from the chloroplasts (Fig. 1).

**Table 1.** Roles of lipophagy and lipolysis in the degradation lipid droplets in yeast, algae, animals, and plants

Organism/model	Mechanism involved in lipid degradation	System/growth conditions	References
*Puccinia distincta*	Micro-autophagy	Ageing of aeciospores	Weber and Davoli (2002)
*Saccharomyces cerevisiae*	ATG-dependent micro-autophagy	Stationary phase	van Zutphen *et al.* (2014); Wang *et al.* (2014)
*Auxenochlorella protothecoides*	Micro-autophagy	Hetero- to auto-trophy	Zhao *et al.* (2014)
*Micrasterias denticulata*	Macro-autophagy	Carbon starvation	Schwarz *et al.* (2017)
*Chlamydomonas reinhardtii*	ATG8- and ATG3-dependent macro-autophagy	Nitrogen starvation	Kajikawa *et al.* (2019)
*Drosophila* larval adipose tissue	ATG8- and Rab32-dependent macro-autophagy	Starvation	Wang *et al.* (2012)
Hepathocyte cells	GTPase dynamin 2 orchestrates autophagy-mediated breakdown of LDs	Starvation	Schulze *et al.* (2013)
Mouse intestinal epithelium	LC3-dependent macro-autophagy	Force-fed with olive oil	Khaldoun *et al*. (2014)
Mouse fibroblasts	Chaperone-mediated autophagy of the LD-associated PLIN2 and PLIN3	Starvation	Kaushik and Cuervo (2015)
Mouse embryonic fibroblasts	Lipolysis by ATGL. Macro-autophagy supplies FAs from membranes	Starvation	Rambold *et al.* (2015)
Hepathocytes	Rab7-dependent lipophagy. Macro-autophagy	Starvation	Schroeder *et al.* (2015); Schulze *et al.* (2017)
Rat hypothalamus neurons, and brown fat tissues and liver	ATGL contains a LC3-interacting region; mutating the LIR motif of ATGL displaces ATGL from LDs and disrupts lipolysis	Cold exposure of *atg7* mutants	Martinez-Lopez *et al.* (2016)
Mouse adipocytes	HSL/ATGL-independent lipolysis. LDs are captured by endo-lysosomal vesicles	Mitochondrial uncoupling drugs	Demine *et al.* (2018)
Hepatocytes of mouse	Down-regulation of PLIN2 stimulates TAG catabolism via autophagy.	*plin2–/–* and PLIN2 over- expressor	Tsai *et al.* (2017)
Tapetum cells of rice	OsATG7-dependent macro-autophagy	Post-meiotic anthers	Kurusu *et al.* (2014)
Arabidopsis seeds	Peroxisome extensions deliver SDP1 lipase to LDs	Seeds	Thazar-Poulot *et al.* (2015)
Arabidopsis seedlings	TAGs and FAs accumulate in *atg5* and *atg7*	6-d-old seedlings in the dark	Avin-Wittenberg *et al.* (2015)
Arabidopsis leaves	Lipolysis by SUGARDEPENDENT1	Extended darkness	Fan *et al.* (2017)
Maize leaves	Disruption of *ZmATG12* affects products of lipid turnover and secondary metabolites	Nitrogen starvation	McLoughlin *et al.* (2018)
Arabidopsis leaves	Macro-autophagy is involved in recycling phospholipids and sphingolipids of the endoplasmic reticulum and plasma membranes	*atg5* and *atg5.sid2* proteomics and lipidomics with or without S and N limitation	Havé *et al.* (2019)
Arabidopsis leaves	Disruption of autophagy slows down membrane lipid turnover in mature and senescing leaves, and FA neo-synthesis in growing leaves. Oleosine1 degradation in the vacuole is impaired in *atg5* mutants. TAGs accumulate in *sdp1* mutants under starvation when autophagy is impaired.	^14^C-acetate labelling and chase in *atg2* and *atg5* mutants combined with *sdp1* lipase mutation or genotypes over- accumulating TAGs	Fan *et al.* (2019)

ATG, autophagy core machinery; ATGL, adipose triglyceride lipase; FAs, fatty acids; LDs, lipid droplets; PLIN2/3, perilipin 2/3 proteins; SDP1, SUGARDEPENDENT1 lipase; TAGs, triacylglycerols.

The role of autophagy in the mobilization of the TAGs stored in the LDs of seeds during the post-germinative phase has not yet been documented. The SDP1 lipase that is associated with the seed LDs is involved in the process of germination (Eastmond, 2006). It is likely that the degradation of the oleosins that coat the LDs is a prerequisite for the mobilization of TAGs (Deruyffelaere *et al.*, 2015). It has been shown that oleosins are marked for degradation by ubiquitination during seed germination. Surprisingly, three distinct and exclusive motifs of ubiquitination can discerned, namely mono-ubiquitin, a K48-linked di-ubiquitin chain, and a K63-linked di-ubiquitin chain, suggesting oleosins may be degraded by both the proteasome and autophagy. Indeed, the ubiquitin–proteasome pathway has been shown to degrade oleosins modified by the addition of a K48-linked di-ubiquitin chain (Deruyffelaere *et al.*, 2015). Proteomic analyses of the LDs of Arabidopsis seeds have revealed that plant UBX-domain containing protein family 10 (PUX10) and cell division cycle 48 homolog A (CDC48A) may be involved in oleosin degradation. Deruyffelaere *et al.* (2018) showed that PUX10 deficiency impairs the degradation of ubiquitinated oleosins and prevents their dislocation from LDs, which precedes the proteasomal degradation of ubiquitinated oleosins. Two studies have independently reported that PUX10 is an integral protein of LDs that recruits CDC48A to the LDs, which suggests that the PUX10–CDC48A interaction may facilitate the dislocation of oleosins from the LDs by the segregase activity of CDC48A (Deruyffelaere *et al.*, 2018; Kretzschmar *et al.*, 2018). Recently, Marshall *et al.* (2019) have described a new class of ATG8-interactors that exploit ubiquitin-interacting motif (UIM)-like sequences for high-affinity binding to ATG8. Interestingly, they found that several PUX proteins (PUX7, PUX8, PUX9, and PUX13) interact with ATG8 using UIM motifs and also interact with CDC48. They also showed that the oleosin OLE1 interacts with ATG8 via an AIM/LIR motif. They consequently proposed that the ATG8-interacting PUX proteins are selective autophagy receptors for CDC48. It remains to be determined whether ATG8 can interact with PUX10 and CDC48A for the degradation of oleosins and LDs by autophagy.

## Conclusions

Interdependent relationships between autophagy and lipid homeostasis are supported by many studies in yeast, algae, and animals. Much less is known about the situation in plants, although some recent advances (see Box 1) suggest a role of autophagy in the degradations of the lipids of the membranes and of LDs (Fig. 1). Further research is required to determine whether and how lipid biosynthesis and LDs play a role in the control of autophagosome formation and in autophagy in plants.

Box 1. **Key developments in understanding the function of plant autophagy in the control of lipid homeostasis**

- **Disruption of basal macro-autophagy impedes lipid turnover and mobilization in the membranes of organelles, and the lipophagy that results in degradation of lipid droplets under carbon stress morphologically resembles yeast micro-autophagy**
Fan *et al.*, (2019) demonstrated that macro-autophagy in Arabidopsis contributes to triacylglycerol synthesis in young leaves, and to lipid turnover from the membranes of organelles in mature and senescing leaves. Lipophagy that is induced in the leaves of Arabidopsis by extended darkness degrades lipid droplets in a process that morphologically resembles yeast micro-autophagy; however, it requires the core components of the macro-autophagy machinery.
- **Lipidomic and proteomic studies of Arabidopsis *atg5* mutants suggest a role of macro-autophagy in the turnover of the plasma membrane, and of the membranes of other organelles except for chloroplasts**
Havé *et al.*, (2019) showed that defects in autophagy trigger ER-stress and increase the abundance of enzymes involved in the metabolism of very-long-chain lipids in the ER and in β-oxidation in peroxisomes. *atg5* mutants exhibit an accumulation of phospholipids and sphingolipids and a decrease of galactolipids compared with the wild-type. This shows that the turnover of lipids originating from the ER and plasma membrane is impaired in the autophagy mutants, while chloroplast and thylakoid membranes may be degraded independently of autophagy.
- **Lipidomic and proteomic studies of maize *atg12* mutants suggest that they have modifications in lipid homeostasis**
  Multi-omic analyses performed by McLoughlin *et al*. (2018) on the leaves of maize autophagy mutants after nitrogen starvation indicated changes in lipids and secondary metabolites that were consistent with results found in Arabidopsis autophagy mutants (Masclaux-Daubresse *et al.*, 2014; Havé *et al.*, 2019).
- **Degradation of ubiquitinated oleosins requires the PUX10-CDC48A protein and is a prerequisite to the degradation of lipid droplets in Arabidopsis seeds**
  The degradation of oleosins is a prerequisite for the post-germination mobilization of the lipids stored in seed lipid droplets (LDs). Deruyffelaere *et al.* (2018) reported that Arabidopsis PUX10 is associated with a subpopulation of LDs during seed germination and is involved in the degradation of ubiquitinated oleosins. PUX10 interacts with ubiquitin and with CDC48A through its UBA and UBX domains, respectively. PUX10 may act as an adaptor that recruits CDC48A to ubiquitinated oleosins, thereby facilitating the dislocation of oleosins from the LDs before their degradation by the proteasome, or by other pathways that remain to be determined.
